# Immunohistochemical Analysis of PD-1 and FOXP3 in Tumor-Infiltrating Lymphocytes in Human Gliomas

**DOI:** 10.7759/cureus.42352

**Published:** 2023-07-24

**Authors:** Priyanka Kanagaraj, Archana Balasubramanian, Raveena Suresh, Bhargavi Somasundaram, Sandhya Sundaram, Priyathersini Nagarajan

**Affiliations:** 1 Pathology, Sri Ramachandra Institute of Higher Education and Research, Chennai, IND

**Keywords:** tils, pd-1, immunohistochemistry, glioma, foxp3

## Abstract

Introduction

Despite the growing advances in molecular research and therapeutics, gliomas continue to be highly invasive and progressive tumors. There is still a need for the development of reliable prognostic biomarkers for effective therapeutic intervention. This study aims to investigate the extent of immunosuppression in glial tumors by analyzing the clinical significance of the expressions of PD-1 and FOXP3 in gliomas.

Methods

This is a retrospective study from 52 glioma patients who underwent surgery. Immunohistochemistry (IHC) for PD-1 and FOXP3 was performed on paraffin-embedded tissue sections manually and their expressions were noted. Data on IDH1 mutational status and mitotic index was collected and statistically analyzed.

Results

Immunohistochemical analysis showed that out of 52 cases, 71.15% (37/52) demonstrated cytoplasmic positivity for PD-1 and 73.1% (38/52) of the cases for nuclear FOXP3 expression. Statistical analysis suggested that elevated PD-1 and FOXP3 expressions were significantly correlated with tumor grade and increased mitotic index (P<0.05 for both the markers).

Conclusion

Concurrent use of checkpoint inhibitors along with other treatment modalities is being studied in a variety of solid tumors. Expressions of negative immune regulators like PD-1 and Foxp3 can pave way for a better understanding of the extent of immunosuppression in the glial tumor environment, which is imperative to formulate new therapeutic approaches.

## Introduction

Gliomas are a heterogenous group of brain neoplasms originating from the glial cells that surround and support the neurons and account for almost 30% of all primary brain tumors, and 80% of all malignant ones, and are responsible for most deaths from primary brain tumors [1]. Conventional treatment options include surgical resection, followed by concomitant radiotherapy and chemotherapy with temozolomide. Despite an improved insight into the underlying molecular mechanisms, it is still challenging to treat patients with high-grade glial tumors and their prognosis remains dismal due to a lack of effective treatment options [2].

Gliomas frequently contain immune cell infiltrates, and several studies have sought to link the degree of infiltration of these cells with survival without conclusively determining how predictive immune cell infiltration inside tumors is [3-5]. Tumor malignancy and treatment outcomes are significantly influenced by the highly heterogenous tumor microenvironment, which is critical in suppressing or enhancing the immune response [6]. Understanding the tumor microenvironment and its mutual effects on the tumor is critical not only for revealing the underlying mechanisms but also for developing novel techniques to increase the efficacy of immunotherapies.

Immunotherapy targeting immune checkpoint proteins such as PD-1/PD L-1 and CTLA-4 has been approved by the FDA for the treatment of a variety of solid tumors [7-10]. However, a comprehensive analysis of the expression of immune checkpoints in brain tumor microenvironment and their roles in tumor progression has not yet been established.

Regulatory T-cells (Treg) are a subset of T-lymphocytes that are CD4+ CD25high and express nuclear transcription factor FOXP3. Treg cells play an important role in maintaining homeostasis by regulating the immune system and mediating self-tolerance [11]. Since FOXP3 is crucial for suppressing anti-tumor immunity, clinical validation of FOXP3 and its associated post-translational modification (PMT) proteins could provide potential therapeutic target and prognostic significance for glioma treatment.

In this retrospective study, we analyzed the infiltration and localization of PD-1 and Foxp3 in Tumor-Infiltrating Lymphocytes (TILs) of archived glioma tissue samples using immunohistochemistry to determine their association with the malignant potential of the tumor and other clinicopathological factors.

## Materials and methods

Study population

In this retrospective case series study, 52 patients with gliomas (astrocytic and oligodendroglial lineages) diagnosed from January 2019 to May 2022 were evaluated. Mixed oligo-astrocytic tumors, ependymomas, non-glial tumors and patients treated with neo-adjuvant therapy were excluded from the study. The clinical records of all patients were reviewed with reference to age, gender, grade at diagnosis, laboratory data, and other clinic-pathological findings. The formalin-fixed, paraffin-embedded specimens were collected from the archives and two qualified neuropathologists re-classified all tumors in accordance with the 2021 WHO categorization system.

TIL evaluation through hematoxylin and eosin (H&E)

TIL infiltration density was classified as mild, moderate, or marked using previously reported semiquantitative grading methods [12], and two institution pathologists did the whole examination through manual eyeballing.

Immunohistochemistry (IHC)

Immunohistochemical analysis was performed using the BenchMark Ventana GX (Roche, Indianapolis, IN, USA) automated immunostainer. Tissue sections (3µm) prepared using the Medimeas (Haryana, India) manual microtome MRM-RM were pre-heated at 75ºC for 10 mins before being deparaffinized with xylenes and then cleared with isopropanol solutions. Heat-induced antigen retrieval was performed using high pH (pH: 9.0) Tris-EDTA buffer. The sections were then incubated with a peroxidase-blocking agent to block non-specific binding followed by incubation with primary rabbit monoclonal anti-PD-1 (Clone: EP239, PathnSitu Biotechnologies, Hyderabad, India) and rabbit monoclonal anti-FOXP3 (Clone: EP340, BioSB, Santa Barbara, CA, USA) antibodies. The antibodies were titrated and standardized using tonsil and normal brain tissue as positive controls. A non-immunological serum replacing the primary antibodies was used as a negative control. After washing, the tissue sections were treated with primary antibodies amplifier followed by incubation with secondary antibody conjugated with HRP molecules (Master Diagnostica, Granada, Spain). Tissue sections were incubated with DAB chromogen, counter-stained with Mayer’s hematoxylin, dehydrated and then mounted.

Scoring

Each section was examined under a light microscope using at least five distinct high-power fields (40 objective and 10 eyepiece) with the most prevalent TIL regions. Cytoplasmic staining for PD-1 and nuclear staining for FOXP3 were determined based on the staining index. Cells at each intensity of staining were graded using a semiquantitative scale of 0 to 3 (0 - no staining, 1 - mild staining, 2 - moderate staining and 3 - intense staining) and so was the proportion of positively stained TILs (0: no positive staining, 1: ≤10%, 2: 11-50% and 3: >50%). The staining index was calculated as follows: staining index = intensity of staining × proportion of positively stained TILs. A total score of 0 was considered as absent staining, 1-2 as low and ≥3 was denoted high staining pattern.

Ethics

· This retrospective study followed the updated premises of the Declaration of Helsinki.

· This study was in accordance to the ethical standards and was approved by the institutional ethical committee (Ref no: CSP/22/JUN/111/335).

Statistical analysis

Statistical analysis was performed using SPSS software version 21 (IBM Corp., Armonk, NY, USA). Pearson’s Chi-square test was used to examine the relationship between PD-1 and FOXP3 expression in TILs and other factors. A p-value of < 0.05 was considered statistically significant.

## Results

Patient characteristics

**Figure 1:**
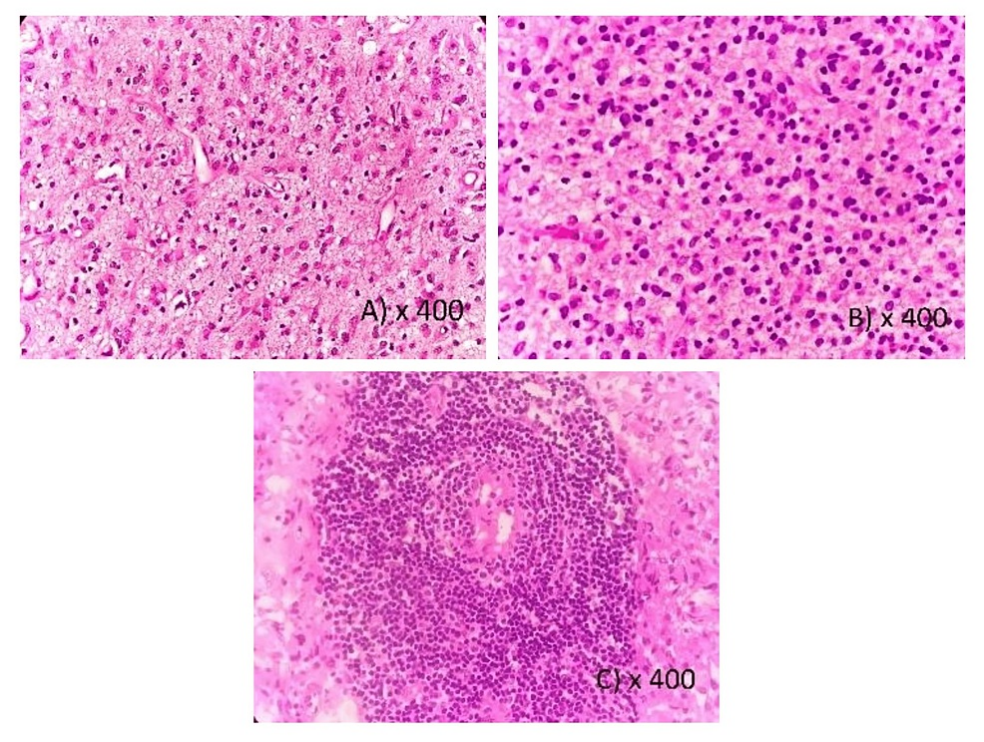
H&E evaluation of TILs: A) Mild TIL density, B) Moderate TIL density, C) Marked TIL density H&E - Hematoxylin and Eosin; TILs - Tumor Infiltrating Lymphocytes

The study population comprised 30 grade-IV astrocytomas, three grade-III anaplastic astrocytomas, two grade-II diffuse astrocytomas and three grade-I pilocytic astrocytomas, 10 grade-III anaplastic oligodendrogliomas and four grade-III diffuse oligodendrogliomas. The tumors were mostly found in the elderly population with a median age of 50.2 years (range: 8-74 years) and males constituted 63.4% of all cases. TIL density was graded as mild (40.3%), moderate (38.4%), or marked (21.1%) (Figure 1). While mild TIL density was predominantly observed in low-grade tumors (66.66%), high-grade tumors prominently exhibited marked lymphocyte infiltration (26.66%). IHC data for the expression of IDH1 in tumor cells were available for 42 cases and Ki67 were available for all cases. The clinicopathology data of the patients are summarized in Table 1.

**Table 1:** Clinicopathological characteristics of the patients (n=52)

Variable	n	%
Age		
< 55 yrs	26	50.0
≥ 55 yrs	26	50.0
Gender		
Male	33	63.5
Female	19	36.5
Tumor location		
Frontal lobe	25	48.1
Temporal lobe	14	26.9
Others	13	25.0
WHO tumor grade		
Grade I	3	5.8
Grade II	6	11.5
Grade III	13	25.0
Grade IV	30	57.7
TIL density		
Mild	21	40.4
Moderate	20	38.5
Marked	11	21.1
Histological lineage		
Astrocytoma	38	73.1
Oligodendroglioma	14	26.9
IDH1 status		
Wildtype	21	50.0
Mutant	21	50.0
Ki67		
Low (<5%)	5	9.6
High (≥5%)	47	90.4

PD-1 expression in gliomas

**Figure 2:**
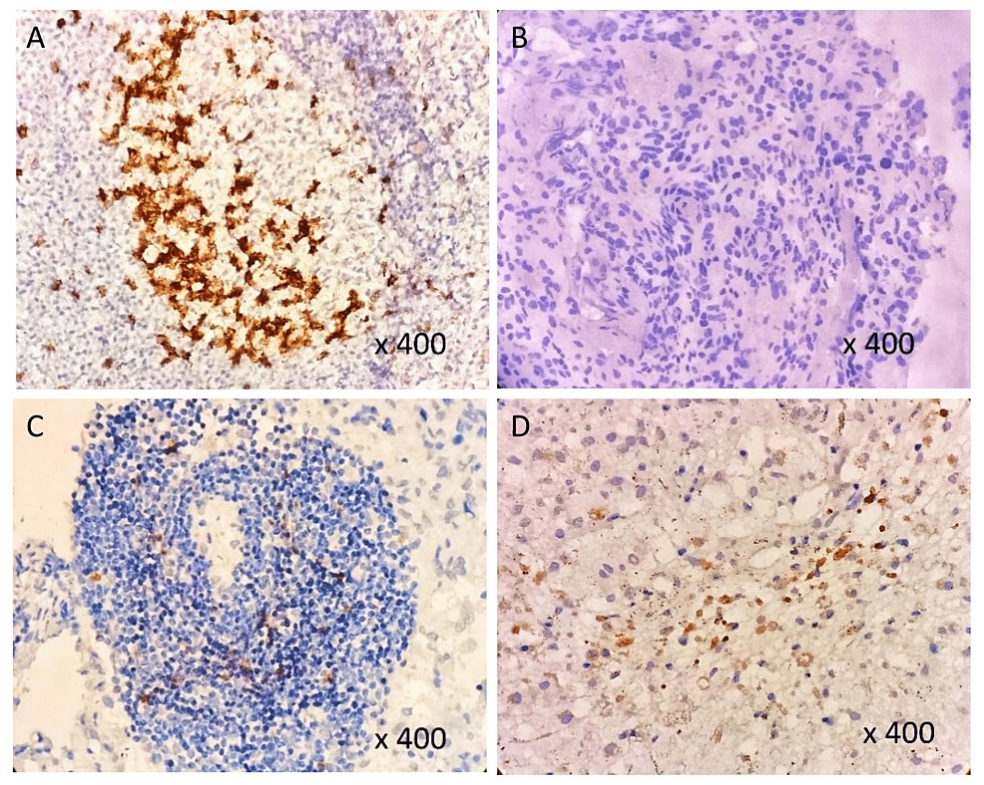
PD-1 expression by IHC: A) Tonsil (positive control), B) No staining, C) Low staining pattern, D) High staining pattern IHC - Immunohistochemistry

**Figure 3:**
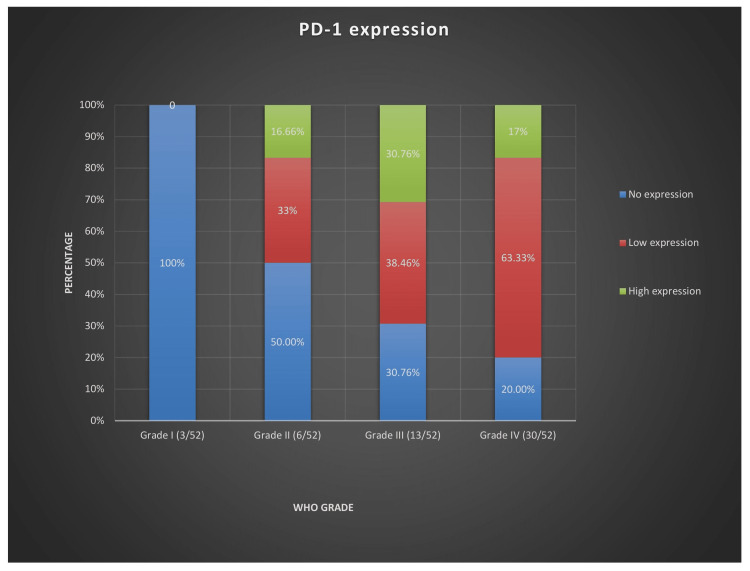
PD-1 expression according to WHO grade

The findings revealed that PD-1 was widely expressed in the cytoplasm (Figure 2A) of tonsil specimens, while the control sample was completely devoid of its expression (Figure 2B). PD-1 was expressed on TILs in 71.15% (37/52) of the cases and its expression was essentially unreported in grade-I samples but low expression was seen in interstitial lymphocytes of grade-II samples, with sparse distribution and common presence in the perivascular region (Figure 2C). The number of cells exhibiting high PD-1 (Figure 2D) expression was significantly larger in high-grade gliomas than in low-grade gliomas (Figure 3), irrespective of the histological lineage, with the majority of cases exhibiting a low level of heterogeneity.

FOXP3 expression in gliomas

**Figure 4:**
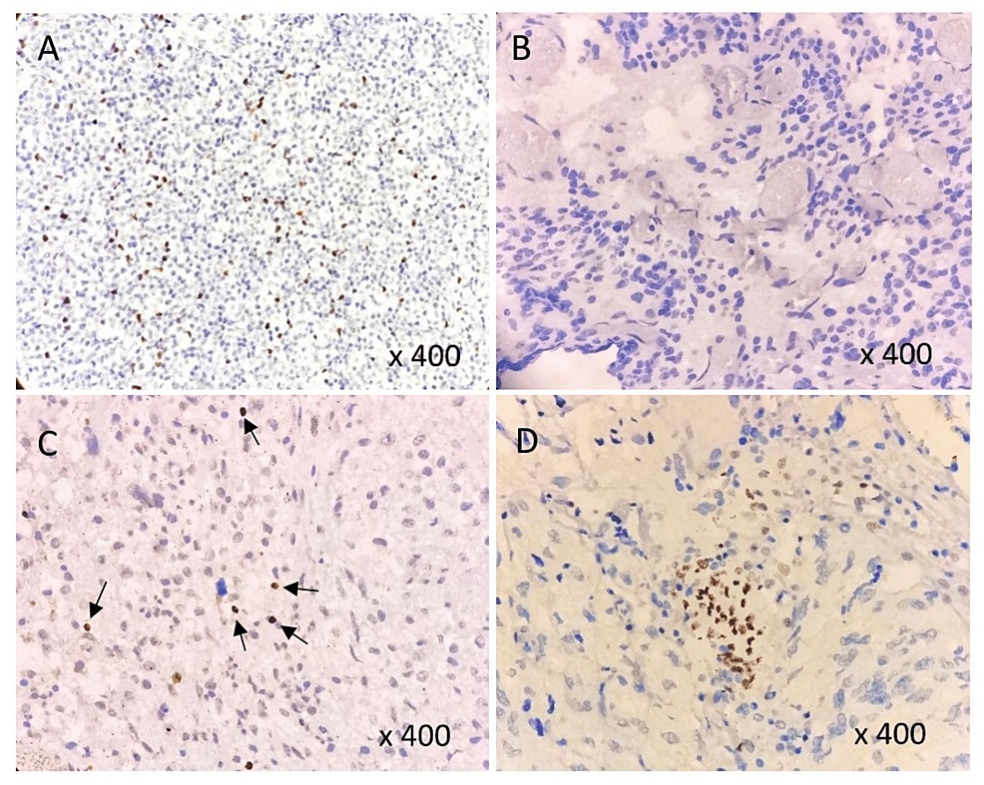
FOXP3 expression by IHC: A) Tonsil (positive control), B) No staining, C) Low staining pattern, D) High staining pattern IHC - Immunohistochemistry

**Figure 5:**
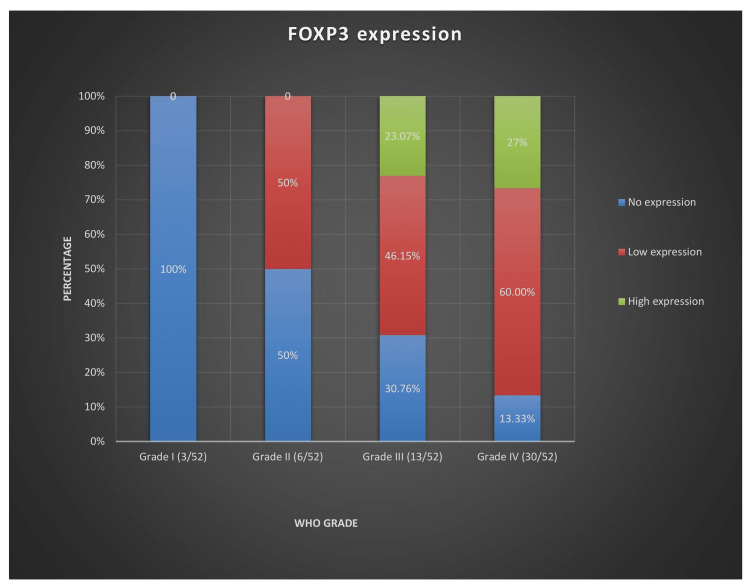
FOXP3 expression according to WHO grade

Nuclear positivity for FOXP3 was demonstrated in 73.07% (38/52) of the cases along with tonsil specimen (Figure 4). While FOXP3 expression was undetected in the control specimen and grade-I tumors, low sporadic expression was observed in 55.81% (24/43) of high-grade tumors. Eleven cases (25.58%) of high-grade gliomas exhibited high FOXP3 expression (Figure 5), with high staining pattern centering the perivascular area in the majority of the cases. Both astrocytic and oligodendroglial subtypes demonstrated FOXP3 positivity, with high expression confining to high-grade tumors.

Correlation between clinical features and immunohistochemical characteristics of the patients

Tables 2, 3 summarize the clinical features and immunohistochemical characteristics of the patients. Positive PD-1 and FOXP3 expressions were shown to be substantially associated with tumor grades (P = 0.036) and (P = 0.009) respectively, but not with age (P = 0.308, for PD-1 and 0.368, for FOXP3), gender (P = 0.12, for PD-1 and 0.171, for FOXP3), tumor location (PD-1 = P: 0.465 and FOXP3 = P: 0.848) or histological lineage. FOXP3 expression was significantly correlated with TIL density (P = 0.002), however, no statistical correlation was observed between marked TIL density and high PD-1 expression.

**Table 2:** Correlation of PD-1 expression with various parameters

Parameters	PD-1						χ2	P-value
	Absent		Low		High			
	n	%	n	%	n	%		
Age							2.349	0.308 Not significant
< 55 yrs	10	38.46%	12	46.15%	4	15.38%		
≥ 55 yrs	5	19.23%	16	61.53%	5	19.23%		
Gender							4.233	0.12 Not significant
Male	7	21.21%	18	54.54%	8	24.24%		
Female	9	47.36%	8	42.10%	2	10.52%		
Location							3.581	0.465 Not significant
Frontal	8	32%	14	56%	3	12%		
Temporal	4	28.57%	5	35.71%	5	35.71%		
Others	4	30.76%	7	53.84%	2	15.38%		
TIL density							8.766	0.067 Not significant
Mild	8	38.09%	12	57.14%	1	4.76%		
Moderate	7	35%	8	40%	5	25%		
Marked	0	0%	8	72.72%	3	27.27%		
Tumor grade							6.610	0.036 Significant
High grade (III, IV)	10	23.25%	24	55.81%	9	20.93%		
Low grade (I, II)	6	66.66%	2	22.22%	1	11.11%		
Histology							0.395	0.820 Not significant
Astrocytomas	11	28.94%	20	52.63%	7	18.42%		
Oligodendrogliomas	5	35.71%	6	42.85%	3	21.42%		
IDH1							0.219	0.895 Not significant
Wildtype	7	33.33%	11	52.38%	3	14.28%		
Mutant	6	28.57%	11	52.38%	4	19.04%		
Ki67							7.153	0.028 Significant
Low (<5%)	4	80%	1	20%	0	0%		
High (≥5%)	11	23.40%	27	57.44%	9	19.14%		

**Table 3:** Correlation of FOXP3 expression with various parameters

Parameters	FOXP3						χ2	P-value
	Absent		Low		High			
	n	%	n	%	n	%		
Age							1.998	0.368 Not significant
< 55 yrs	9	34.61%	13	50%	4	15.38%		
≥ 55 yrs	5	19.23%	14	53.84%	7	26.92%		
Gender							3.526	0.171 Not significant
Male	6	18.18%	19	57.57%	8	24.24%		
Female	8	42.10%	8	42.10%	3	15.78%		
Location							1.375	0.848 Not significant
Frontal	8	32%	11	44%	6	24%		
Temporal	3	21.42%	8	57.14%	3	21.42%		
Others	3	23.07%	8	61.53%	2	15.38%		
TIL density							17.075	0.002 Significant
Mild	8	38.09%	12	57.14%	1	4.76%		
Moderate	6	30%	11	55%	3	15%		
Marked	0	0%	4	36.36%	7	63.63%		
Tumor grade							9.412	0.009 Significant
High grade (III, IV)	8	18.60%	24	55.81%	11	25.58%		
Low grade (I, II)	6	66.66%	3	33.33%	0	0%		
Histology							0.853	0.652 Not significant
Astrocytomas	9	23.68%	21	55.26%	8	21.05%		
Oligodendrogliomas	5	35.71%	6	42.85%	3	21.42%		
IDH1							0.681	0.711 Not significant
Wildtype	6	28.57%	12	57.14%	3	14.28%		
Mutant	6	28.57%	10	47.61%	5	23.80%		
Ki67							8.044	0.018 Significant
Low (<5%)	4	80%	1	20%	0	0%		
High (≥5%)	10	21.27%	26	55.31%	11	23.40%		

Molecular associations of IDH1 mutations and Ki67 with PD-1 and FOXP3 expressions

IDH1 mutations were not associated with either PD-1+ TIL or FOXP3+ TIL expression (P = 0.895 for PD-1 and P = 0.711 for FOXP3), regardless of the glioma's grade or pathology. Despite PD-1/FOXP3 expression remaining independent of the IDH1 mutational status of the tumor, the significant association of high PD-1 and FOXP3 expressions with high mitotic index (P = 0.028 for PD-1) and (P = 0.018 for FOXP3) suggests that elevated PD-1 and FOXP3 expressions may be linked to the progression of glial tumors.

## Discussion

The investigation of immunological markers at the tumor site, particularly TILs, is critical for the development of effective immunotherapy [13]. Even though the brain is an immunologically privileged organ, substantial case series have shown lymphocytic infiltration into gliomas [3,14]. It is unknown, however, whether these TILs in glioma contribute to host immunosurveillance [15] and patients' responses to combination treatment or if they take part in cancer immunoediting and tumor-specific immunosuppression [16]. Their function in gliomas may be partially indicated by the extent of lymphocyte infiltration and the prognostic value of TILs.

The downregulation of immune responses is a crucial function of PD-1 and FOXP3, which may have an impact on the gliomagenesis. In this study, we systematically investigated the expression profiles of PD-1 and FOXP3 in TILs of astrocytic and oligodendroglial lineages of gliomas. Mild and moderately infiltrating TILs were observed in the majority of the cases. Consistent with this study, Berghoff et al. [17] reported sparse to moderate density of TILs in 72.6% of the cases.

A previous study by Han et al. [18] in 2017 and Garber et al. [12] in 2016 reported that PD-1-expressing tumor-infiltrating cells and grade IV gliomas were significantly correlated. In another study by Wei et al. [19] which investigated the association of PD-1 upregulation with disease progression, it was reported that PD-1+ CD4+ and PD-1+ CD8+ TILs were significantly increased in grade III and IV gliomas. These results were concordant with our observation where persistent expression of PD-1 was observed in high-grade gliomas and there was a significant correlation between WHO grade and PD-1 expression.

FOXP3 expression in our study significantly correlated with the WHO grade and was predominantly expressed in high-grade gliomas. This is consistent with Wang et al.’s [20] study which demonstrated that FOXP3 upregulation is significantly correlated with histologic grades of gliomas. Previous studies of ectopic expression of FOXP3 in tumor tissue and cancer cell lines resulted in the observation that aggressiveness and immune evasion of tumors are correlated with FOXP3 expression (Hinz et al. [21]; Cunha et al. [22]). These findings suggest that FOXP3 may operate as a tumor promoter in some types of cancers. The link between FOXP3 expression and patient prognosis, on the other hand, remains debatable. Ladoire et al. [23] discovered that FOXP3 expression was an independent predictive factor for increases in both relapse-free and overall survival in HER2-over-expressing breast carcinomas. Merlo et al. [24] on the other hand, discovered that FOXP3 expression in tumors was adversely related to patient survival. These researchers also discovered a link between FOXP3 expression and lymph node metastases, suggesting that FOXP3 expression was associated with a poor prognosis.

A study published by Heimberger et al. [25] reported significant differences in the prevalence of FOXP3+ Tregs between tumors of different grades and histologic tumor subtypes, and demonstrated that FOXP3+ Tregs were most frequently expressed in glioblastoma multiforme (GBM), but very rarely in low-grade astrocytomas. In line with the reports (Hinz et al. [21]; Cunha et al. [22]), we discovered that FOXP3 was not expressed in normal brain tissues and sporadic expression can be identified in the tumor cells of grade III gliomas, and greater expression, particularly in the nucleus of tumor cells, may be seen in GBM tumors.

Mutations in IDH1 gene are prominently observed in a high percentage of grade II and III tumors of both astrocytic and oligodendroglial lineage as well as secondary GBM. Previous studies have reported that IDH1 mutations associated with younger age had increased overall survival in patients diagnosed with glioma [26-28]. Because of its prognostic significance, IDH1 has emerged as a therapeutic target and IDH1 inhibitors are being studied in solid tumors including gliomas (NCT 02073994, NCT 02381886). The lack of association between PD-1/FOXP3 with IDH1 mutational status of the tumor is probably one of the most fascinating findings of our study, which suggests that only a highly select subset of patients will benefit from combinatorial therapeutic options.

We found both PD-1+ and FOXP3+ lymphocytic infiltration to be associated with increased Ki67 expression, which is a tumor proliferative activity marker and a prognostic indicator in a number of solid malignancies. In agreement with our results, Mitchell et al. [29] demonstrated that biologically aggressive non-small cell lung cancer is linked to higher Ki67 expression, which is also linked to higher immune checkpoint expression and lower intra-tumoral immune cell infiltration.

The positive correlations between both PD-1 and FOXP3 levels with tumor grade and Ki67 in gliomas appear to reinforce the finding that their expression may be correlated with a poor patient prognosis. However, patients’ survival data and the association with other prognostic markers must be comprehensively analyzed to accurately assess the overall incidence and establish the reliability of the prognostic significance of PD-1 and FOXP3.

## Conclusions

Our results imply that PD-1 and FOXP3 expressions in glioma infiltrating lymphocytes play a significant role in the progression of the tumor into high-grade neoplasm and may be linked to the biological malignancy of high-grade glial tumors. The findings regarding the glioma immune environment also point to the possibility of immune checkpoint inhibitors being successful in our population. The results also suggest it is possible that both PD-1 and FOXP3 will emerge as novel glioma molecular markers and therapeutic targets. Further research is necessary, nevertheless, to fully understand their obvious biological role in glioma cells. Further confirmation by molecular studies and survival data of the patients is warranted with a larger sample size.
